# Epilepsy, in which Castration Was Performed

**Published:** 1859-08

**Authors:** 


### EPILEPSY, IN WHICH CASTRATION WAS PERFORMED.

Mr. C. Holthouse read before the Royal Medical and Chirurgical Society (March 22, 1859) an account of a remarkable case of epilepsy, in which he castrated the patient, an operation which has excited much interest and comment abroad, and which we cannot but consider as indefensible. The author commenced his paper by citing ten cases of epilepsy, in which castration is said to have cured the disease. In the first case, the testicles were removed in consequence of disease of those organs. In the second case, one testicle was removed on account of an accident to the organ. Both patients were epileptic previous to the affection of the testicles; and their removal, though not done with the view of curing the epilepsy, incidentally did so. These cases were related to Dr. M'Kinley, of Tennessee, U. S., by a Mr. M'Gavoc, of the British Navy, and are published in the *American Medical Gazette*, July, 1855, along with seven other cases in which castration was performed for the express purpose of curing epilepsy. Of these seven cases, two occurred in the practice of Dr. M'Kinley himself; two in the practice of Dr. Talbot, of Missouri; and one of that of Dr. Hacker, of Louisiana. The tenth case occurred in Germany, the operation having been performed by Holz, under the direction of Joseph Frank, and it is recorded in the *Praxeos Medicæ Universæ Precepta*, vol. ii. chap. 11. Mr. Holthouse stated that he brought his case before the notice of the society partly because the operation was not attended with the same favorable results as in the cases referred to, and partly for the purpose of removing the misapprehension which prevails in reference to it. The case of the patient, very nearly as taken by Mr. Ponsonby K. Adair, the house-surgeon of the Westminster Hospital, was then read; after which the author proceeded to state the grounds on which he was induced to operate in the following words: "Reasons which induced me to operate.—1. The patient's urgent request. 2. The simple and dangerless character of the operation. 3. The knowledge that epilepsy had been cured by castration. 4. The possibility, if not probability, that it might be in the present case. Lastly, I was further influenced by a consideration of the history of the case, which showed, first, that every remedy hitherto tried had failed; and, secondly, that there was a close connection between the origin and severity of the fits and the condition of the sexual organs. Whatever weight may be attached to the 2d, 3d, and 4th reasons, I confess I should not have felt myself justified in recommending a proceeding of which I had no personal experience, nor any knowledge of the individuals who had adopted it; but although these considerations would not have sufficed to make me recommend the operation, they had their influence in inducing me to consent to its performance. It will be obvious, then, that I was chiefly influenced by the wishes of the patient. The question, therefore, arises, how far may a patient's wishes as respects treatment be acceded to, and on what point must a physician or surgeon satisfy himself before acting on a patient's suggestion? The following seem to me to be essential: 1st, he must satisfy himself that the patient is of mature age; 2dly, that he is of sound mind, not the subject of hallucinations or a monomaniac; 3dly, that his proposition is not unreasonable; and lastly, that the remedy proposed is not a dangerous one. Now, the only points on which I apprehend there can be any difference of opinion are—1st, as to the sanity of the patient; and 2dly, as to the reasonableness of the treatment adopted. I shall, therefore, address myself to these two points. And first, as to the sanity of the patient, it has been asserted by many that the mere fact of the man having had epilepsy for so long a period was presumptive evidence that his mind was unsound, and the pertinacity with which he begged to be castrated was considered a sufficient proof that this opinion was not unfounded. Mania, I was reminded, sometimes takes this form, and lunatics have been known to castrate themselves. Now, I am free to admit that if such a desire existed, or such a request were made on the part of any individual without adequate motive, there would be legitimate grounds for believing that he was insane, or at least, laboring under an insane delusion. But was there no adequate motive in the present case? What are the facts? The patient is walking in one of the streets of New York, when he is stopped by a physician of eminence and repute, the editor of a medical journal, who informs him he has just received a communication from one of his correspondents, in which castration is recommended for certain forms of epilepsy attended with great venereal excitement, and that nine cases had been successfully treated by this method. The letter containing this announcement Dr. Reese reads to him, and advises him to submit to the operation, at the same time offering him an introduction to Prof. Parker, Surgeon of the Bellevue Hospital, and Professor of Surgery at the University of New York, and one of the most eminent surgeons in that city. A consultation is held between Dr. Reese and Prof. Parker, when the latter having been made acquainted with the history of the case, and had his attention called to the cases published in Dr. Reese's journal, consents to perform it, and is only deterred from doing so by the interposition of a medical relative of the patient, who was of opinion that no benefit would result. From this period is to be dated the desire of the patient to be castrated; and believing that he had at last found a remedy for his disease, is it to be wondered at that he should be earnest and urgent in his desire to avail himself of it? Moreover, when he called to mind the origin of his fits—their recurrence on his first marriage, after they had been absent for the two preceding years—their exacerbation on his second marriage, as well as under any sexual excitement—and his frequent nocturnal pollutions—could he dissociate the fits from the sexual organs? And was not the fact of his thus associating them a proof rather of his sanity than of his insanity? I appeal to the candor of the society whether there is the least analogy between a man desiring castration under such circumstances, and the morbid craving for mutilation of a madman? I would further remark, that the removal of the exciting cause of epilepsy, as indeed of any other disease, has been long recognized as the established rule of practice; and, although it is not always possible to determine what this cause may be, I hold it to be generally admitted that we are justified in acting on a fair presumption of the cause, though such presumption must necessarily fall short of actual proof. Acting on this presumption, epileptic patients have been subjected to the dangerous operation of trephining, sometimes with and sometimes without success. Acting on such presumption, Wardrop cured a case of eccentric epilepsy, by removing a joint of a healthy finger. Tissot relates a case where a similar good result followed amputation of the great toe. Dr. W. H. Edwards, of Virginia, U. S., cites another, in which the leg was amputated a few inches below the knee-joint; and several similar or analogous examples are on record. It must be obvious, then, that in all of these cases the epilepsy was of eccentric origin, and that the great nervous centres were only affected secondarily and sympathetically. Now, the whole history of the patient whose case forms the subject of this paper, pointed to the abuse of the reproductive organs as the original source and the subsequent exciter of the epileptic paroxysms; indeed, the early abuse to which they had been subjected had so increased their functional activity that they were habitually in a state of abnormal excitement, and this reacting on the brain prevented that repose of the organ so favorable, if not essential, to its recovery, and constantly tended to counteract the effect of other remedial agents. But I am assuming here that the brain is affected; and indeed, in so long standing a case, it would be absurd to suppose that this organ was not in some measure damaged, although it may not originally have been the starting-point of the disease. I am aware it may be objected that the excitement of the genital organs was the effect and not the cause of disease of the brain, and that the origin both of the fits and of the sexual excitement must be referred to cerebral disorder. Now, though it may not be possible to prove the negative of this, there are so many facts which demonstrate the influence of the reproductive organs over the cerebral functions, that it is no unfair inference to suppose they may have thus acted in the present case. I hold, then, that the removal of all extrinsic sources of excitement from a disordered brain is both reasonable and proper; and believing, as from the history of the case I was entitled to do, that the reproductive organs of this patient did exercise an injurious influence on his brain—seeing, moreover, that precedents were not wanting where the removal of the testicles, under like circumstances, had been attended with success—I say, taking into consideration all these circumstances, I maintain that the request of the patient was not unreasonable, and that it was perfectly justifiable to accede to his wishes. The results of the operation are, so far as can be judged of at present, certainly not such as the patient anticipated: the fits continue to recur with much the same frequency, and are of a similar character."

Mr. Solly said he saw the patient in St. Thomas' Hospital, where a consultation was held, and Mr. Simon was inclined to yield to the man's wishes, against the general feelings of his colleagues. He (Mr. Solly) suggested, that if any operation was performed, the spermatic artery should be tied on one side, and if that proved in any way successful, a similar operation might be performed on the opposite side. His reason for making the suggestion was, that castration was by no means a harmless operation, or unattended with danger. The last patient whom Mr. Green castrated died within a short time after the operation.

Mr. Holmes Coote said that the patient, having enjoyed the hospitalities of the public institutions in France and Germany, might now be seen any day in St. Bartholomew's walking about and airing himself, in the conscious dignity of having been castrated. It appeared to him that the man was a monomaniac. In an institution with which he was connected, Bethlehem Hospital, if all the operations were performed which the patients desired there would be a strange collection of individuals. One man desired to have his throat cut; another wished to be hung, and a third cut off his penis. In the case of a young woman subject to erotic monomania, a surgeon consented, at her request, to excise the clitoris, with the same effect as in Mr. Holthouse's case. The disease was really in the brain, and could only be relieved in an asylum under proper supervision.

Dr. Schuloff stated that eunuchs in the east were found to suffer from epileptic fits.

Dr. Webster had no doubt that Mr. Holthouse's patient was of unsound mind, and the circumstance of his strong desire to be castrated he regarded as a striking indication of that fact. He did not think the surgeon was justified in performing the operation simply because the patient requested it. The statement of the American cases, however, appeared to be sufficient to justify the operation. Long-standing epilepsy was generally accompanied with unsoundness of mind.

Mr. Acton said there were many instances recorded of *operations de complaisance*, so that Mr. Holthouse was not without precedents for his justification. He hoped, however, that surgeons would be prevented from performing an operation of the kind detailed, seeing that the results were so unsatisfactory. He wondered that no one appeared to have suggested the cauterization of the urethra, which some persons considered useful in such affections.

Mr. Hale Thompson had seen the patient in Westminster Hospital, and had no doubt of his insanity. Had he heard of cauterization no doubt he would have proposed it. He had heard more false logic in the paper than he had ever heard in the society before; and he suggested whether it would not be better to follow the rules of practice laid down by eminent surgeons in this country, rather than adopt ill-understood American eccentricities. When the operation was performed he (Mr. Thompson) protested against it, the testicles being perfectly sound, and left the theatre rather than sanction by his presence such a proceeding. The operation was not without danger, and the man suffered fearfully from hemorrhage for two hours.

Dr. Ogle believed the man to be a monomaniac, and he had seen a letter from Paris respecting him, in which it was stated "the physicians think him stark mad." With regard to the experiments upon the lower animals, it was found that if they were castrated before puberty, their sexual desire was entirely destroyed, but was not the case if the operation was performed at a later period. Cases were on record in which children had been begotten after castration, and it was known that some eunuchs had harems of their own; and it had been stated that a monarch of Persia, finding that the removal of the testes from eunuchs did not destroy desire, ordered that the entire organ should be removed. Juvenal, also, in his sixth satire, had a passage in which he showed that the women were in the habit of indulging themselves with eunuchs.—*Med. Times and Gazette*, April 2, 1859.

